# Bioactive glass selectively promotes cytotoxicity towards giant cell tumor of bone derived neoplastic stromal cells and induces MAPK signalling dependent autophagy

**DOI:** 10.1016/j.bioactmat.2022.02.021

**Published:** 2022-02-28

**Authors:** Joerg Fellenberg, Sarina Losch, Burkhard Lehner, Marcela Arango-Ospina, Aldo R. Boccaccini, Fabian Westhauser

**Affiliations:** a Center for Orthopedics, Trauma Surgery and Spinal Cord Injury, Heidelberg University Hospital, Heidelberg, Germany; b Institute of Biomaterials, Department of Materials Science and Engineering, University of Erlangen-Nuremberg, Germany

**Keywords:** Bioactive glass, Giant cell tumor of bone, Mesenchymal stromal cells, Mitogen-activated protein kinases, Cytotoxicity, Autophagy, GCTB, giant cell tumor of bone, GCTSC, giant cell tumor derived stromal cell, BMSC, bone marrow derived stromal cell, BG, bioactive glass, MAPK, mitogen activated protein kinases

## Abstract

Giant cell tumors of bone (GCTB) are associated with massive bone destructions and high recurrence rates. In a previous study, we observed cytotoxic effects of three different compositions of bioactive glasses (BGs) towards GCTSC but not bone marrow derived stromal cells (BMSC) indicating that BGs represent promising candidates for the development of new therapeutic approaches. In the current study we aimed to investigate the molecular mechanisms that are involved in BG induced cytotoxicity. We observed, that BG treatment was not associated with any signs of apoptosis, but rather led to a strong induction of mitogen activated protein kinases (MAPK) and, as a consequence, upregulation of several transcription factors specifically in GCTSC. Genome wide gene expression profiling further revealed a set of fifteen genes that were exclusively induced in GCTSC or induced significantly stronger in GCTSC compared to BMSC. BG treatment further induced autophagy that was significantly more pronounced in GCTSC compared to BMSC and could be inhibited by MAPK inhibitors. Together with the known osteogenic properties of BGs our findings support the suitability of BGs as therapeutic agents for the treatment of GCTB. However, these data have to be verified under in vivo conditions.

## Highlights

• Bioactive glasses (BG) are selectively cytotoxic towards neoplastic stromal cells.
• BG induced cell death is independent from apoptosis.
• BG activates mitogen activated protein kinases and transcription factors.
• BG trigger differential gene expression in neoplastic versus normal cells.
• BG induce autophagy.

## Graphical abstract

**Figure:**
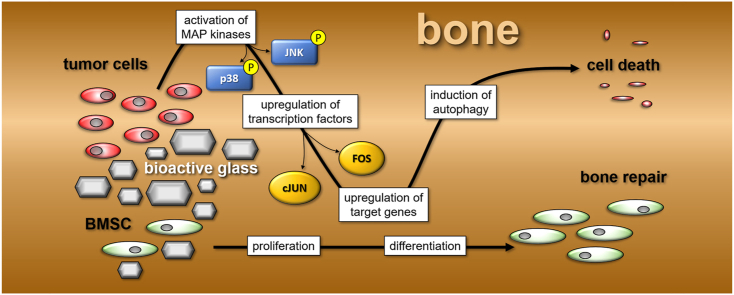

## 1 Introduction

Giant cell tumor of bone (GCTB) is a semi-malignant primary bone tumor, typically affecting the meta-epiphyseal regions of long bones with a peak incidence between 20 and 40 years of age [1]. GCTB is characterized by an unpredictable biological behavior with locally aggressive growth. As a result, expansive osteolytic defects associated with significant bone destructions are very common caused by the resorptive activity of reactive osteoclast-like multinucleated giant cells found in large numbers in GCTB. Attraction of osteoclast precursors and activation of osteoclasts is triggered by the production of RANKL (receptor activator of NF-κB ligand) and various chemokines by the neoplastic GCTSC population [2,3]. GCTBs are characterized by a glycine to tryptophan substitution at position 34 in the H3F3A gene encoding the histone variant H3.3 [4]. The mutation is supposed to occur in differentiating mesenchymal stem cells resulting in massive epigenetic alterations and the induction of an impaired osteogenic differentiation [5].

The primary treatment option for GCTG is surgery. Tumor resection with curettage and joint preservation is most often preferred, although this approach is associated with very high recurrence rates up to 65% [6,7]. The use of several toxic or thermal adjuvants like liquid nitrogen, alcohol, phenol, hydrogen peroxide and polymethylmethacrylate (PMMA) has been suggested to reduce local recurrence rates. However, results are inconsistent and unwanted side effects like local tissue damage and hepatotoxicity have been reported [[8], [9], [10], [11]]. Further, a systemic therapy approach with denosumab, an antibody directed against RANKL has been introduced in order to block RANKL action, thus minimizing giant cell formation and activity. However, this strategy does not target the neoplastic stromal cells and has been discussed controversially because of unclear benefits and even a potentially increased risk of new malignancies due to immunosuppression [12,13]. A recent review of the literature revealed that the use of denosumab induces positive but variable histological responses with several types of adverse effects. Concerning the oncological outcome, no effects on metastatic disease nor local recurrence rates could be observed [14]. Thus, improvement of GCTB therapy especially with respect to the reduction of tumor recurrence rates is still an urgent clinical concern.

Bioactive glasses (BG) are in most cases silica (SiO_2_)-based, non-crystalline inorganic biomaterials with varying amounts of different components like sodium oxide (Na_2_O), calcium oxide (CaO) and phosphorus pentoxide (P_2_O_5_), which were invented 1969 [15]. During further BG development also borate and phosphorus-based compositions were established and additional ions like zinc, magnesium or strontium were included into the glass composition in order to alter their functionality and bioactivity, for example for the local delivery of therapeutic bioactive ions. The outstanding properties of BGs are their biocompatibility, their bioactivity mediated by ionic dissolution products, their antibacterial behaviour and their ability to form strong bonds to the surrounding hard and soft tissue based on the formation of a hydroxycarbonate apatite (HCA) layer on the surface after contact to body fluids [[16], [17], [18]]. BGs have further been shown to promote osteoblast binding, proliferation, differentiation and mineralization, as well as osteogenic differentiation of mesenchymal progenitor cells [[19], [20], [21]]. Together with the documented angiogenic properties [22] these attributes formed the basis for their extensive application in bone and soft tissue engineering [23]. Because of these positive properties, BGs have been employed as implant material for different clinical applications since the approval of 45S5-BG by the food and drug administration (FDA) in 1985 [15,24]. Besides these numerous positive effects, BGs have also been shown to negatively influence their local microenvironment, particularly in the early stages of degradation that is associated with an increased release of ions, such as Na^+^ and Ca^2+^ and significant changes in the pH of the fluid surrounding the BG.

So far, the underlying mechanisms of BG induced cytotoxicity are not yet fully understood and different mechanisms are being discussed in the literature. Mostly, changes in local pH caused by a burst release of BG dissolution products in the early phases of contact to surrounding fluids are considered to be responsible for BG-mediated cytotoxicity [25]. Based on this observation, preconditioning approaches have been evaluated to compensate the initial burst release of ions and thereby enhance the biocompatibility of BGs for cell culture studies [26]. Alternatively, BGs with reduced alkali content have been developed in order to reduce the local pH alteration (increase) and to enhance biocompatibility [27]. Recently, beyond the role of pH changes, also other mechanisms for BG-mediated cytotoxicity were discussed. For example, Yan and co-workers attributed BG-mediated cytotoxicity to an increase in membrane fluidity caused by the ionic dissolution products (mainly Si-ions) from BGs [28]. However, there is increasing evidence that the impact of local pH alterations as well as the presence of the ionic dissolution products on cell viability might be overestimated as also shown in a recent study conducted by our group [29]. In this previous study we identified a selective cytotoxic effect of BGs towards the neoplastic stromal cell population of GCTB (GCTSC) whilst the viability of bone marrow derived stromal cells (BMSC) remained unaffected or was even enhanced in the presence of BGs. This effect was independent from the BG induced pH-shift and dissolution products, but it was shown to be related to the direct contact of cells with BGs [29]. Based on the observed properties, we hypothesized that BGs might be promising biomaterials that could be used for the reduction of GCTB recurrence rates through killing of tumor cells that remained after surgery and the simultaneous promotion of BMSC-mediated bone regeneration due to their known osteogenic properties. The aim of this study was to investigate the therapeutic feasibility of this hypothesis by analyzing the molecular mechanisms underlying the observed cell-specific effects of BGs. The knowledge of these mechanisms might facilitate a potential clinical use of BGs for the treatment of GCTB.

## 2 Materials and methods

### 2.1 Study ethics and cell origin

Primary cell lines were isolated from tissue samples obtained from patients who underwent surgery at the Heidelberg Orthopedic University Hospital. Written informed consent has been obtained from all donors. The study has been approved by the Ethical Committee of the Medical Faculty of the University of Heidelberg. (S-082/2019, S-340/2018) and has been carried out in accordance with the Code of Ethics of the World Medical Association (Declaration of Helsinki). The osteosarcoma cell line HOS143B was used as control (Sigma-Aldrich #91112502).

### 2.2 BG production

The chemical compositions of the BGs used in this study are presented in Table 1. The glasses were produced by the melt-quenching method, as detailed elsewhere [29]. The BGs underwent a sintering process that resembles the heat treatment necessary to shape the materials into 3D structures. The used sintering programs were 1.5 h at 690 °C for ICIE16-BG and 3Zn-BG and 2 h at 1050 °C for 45S5-BG, partial crystallization of the particles was induced as was demonstrated in a previous study [29]. The morphology of the BG particles was characterized by scanning electron microscopy (SEM, Auriga, Carl-Zeiss, Germany) with an accelerating voltage of 1.5 kV. The particle size was estimated from the SEM images measuring at least 100 particles of each BG powder using the software ImageJ (National Institutes of Health Bethesda, MD, USA). Energy-dispersive X-ray spectroscopy (EDX, Oxford Instruments) was used to obtain the elemental composition of the BGs qualitatively and to discard the influence of impurities resulting from the BGs processing.

**Table 1:** Chemical compositions of the BGs used for the experiments.

Chemical composition (mol %)						
BG name	SiO_2_	CaO	Na_2_O	P_2_O_5_	K_2_O	ZnO
**45S5**	46.14	26.91	24.35	2.60		
**ICIE16**	49.46	36.27	6.6	1.07	6.6	
**3Zn**	49.46	33.27	6.6	1.07	6.6	3.0

### 2.3 Isolation of primary cells and cell culture

Bone marrow from patients that underwent primary hip arthroplasty was used for the isolation of BMSC. Isolated bone marrow was fractionated on a Ficoll-Paque Plus density gradient (GE Healthcare Europe, Freiburg, Germany) before the mononuclear cell fraction containing the BMSCs was separated, washed with phosphate-buffered saline (PBS) (Thermo Fisher) and cultured in gelatinized (0.1%) cell culture flasks at 37 °C and 5% CO_2_ in a humidified atmosphere. Culture medium consisted of Dulbecco's modified Eagle's medium (DMEM) (Sigma-Aldrich) high glucose supplemented with 12.5% fetal calf serum (FCS) (Biochrom, Berlin, Germany), 1% non-essential amino acids (NEAA) (Sigma-Aldrich), 50 μM β-mercaptoethanol (Sigma-Aldrich), 100 μg/ml penicillin/streptomycin (Sigma-Aldrich) and 4 ng/ml fibroblast growth factor 2 (Merck-Millipore) Non-adherent cells were removed 24 h after plating while adherent cells were further expanded and passaged at 80% confluency.

For the isolation of GCTSC, tumor tissues were cut into small pieces using scalpels, washed with PBS and digested with 1.5 mg/ml collagenase B (Thermo Fisher) for 3 h at 37 °C diluted in culture medium consisting of DMEM high glucose supplemented with 10% FCS (Biochrom) and 100 U/ml penicillin/streptomycin (Sigma-Aldrich). After the digestion cells were washed twice in PBS and seeded in culture medium. After the first 24 h of culture, cells were treated with Trypsin/EDTA (Sigma-Aldrich) and the stromal cells were transferred to a new culture flask separating them from the giant cells that remained attached. After 3 passages the stromal cell population was free of remaining giant cells and histiocytes.

### 2.4 Cytotoxicity assay

Cytotoxicity of BGs was quantified using a water-soluble tetrazolium salt (WST-1) assay (Roche Diagnostics, Mannheim, Germany). One hundred μl of 2-fold concentrated BGs resuspended and vortexed in cell culture medium were pipetted into each well of a 96-well cell culture plate before 10,000 cells were added to each well. After the desired incubation time the medium was replaced by 100 μl WST-1 reagent diluted 1:10 in cell culture medium and incubated for 120 min at 37 °C. Finally, the optical absorbance of the supernatants was determined in a plate reader (Autobio-Phomo, Anthos Microsystems, Friesoyte, Germany) at 450 nM with a reference wavelength of 600 nM. Wells without cells were used as blanks and subtracted from the experimental sample wells. All measurements were done in triplicates.

## 3 Apoptosis antibody array

Expression of apoptosis related proteins was analyzed using a human membrane-based antibody array (Ray Biotech, Inc., Norcross, GA) covering 43 apoptotic factors (Supplemental Fig. 1). The array was processed according to the manufacturer's instructions. In brief, GCTSC were co-cultured with 45S5-BG (0.5 mg/ml) and proteins were extracted using RIPA lysis buffer (Santa Cruz biotechnologies, Heidelberg, Germany) supplemented with protease inhibitor cocktail (Sigma Aldrich, Munich, Germany) after 12, 24 and 48 h. Cells cultured without BG were used as control. A BCA assay (Pierce, Rockford, USA) was used to quantify protein concentrations. For each membrane 300 μg total protein dissolved in 1 ml blocking buffer were used for each membrane. After an incubation for 30 min in blocking buffer, membranes were incubated with the protein samples overnight at 4 °C. After washing, membranes were incubated with a biotinylated antibody cocktail for 2 h at room temperature, washed and incubated with horseradish peroxidase conjugated streptavidin for 2 h at room temperature. After a final washing step, membranes were incubated with a chemoluminescence substrate, before signals were detected using the gel documentation system Fusion-SL 3500 and quantified using BIO-1D software version 15.01 (Vilber Lourmat, Eberhardzell, Germany). The background signal was subtracted from the obtained values and the resulting values were normalized to the positive controls spotted on each membrane.

### 3.1 Western blot analysis

For LC3 detection 2.5 μg of total protein was separated on 15% polyacrylamide gels and transferred to nylon membranes (Immobilon-P, Millipore, Schwalbach, Germany). For all other antigens 10 μg of total protein and 10% polyacrylamide gels were used. Membranes were blocked with 3% skim milk (Sigma-Aldrich) diluted in PBS supplemented with 0.05% Tween 20 (Sigma-Aldrich) before they were incubated with the primary antibody. The following primary antibodies were used at the indicated dilutions: Actin (1:5000) (BD Biosciences, Heidelberg, Germany), Akt, p-Akt, Erk1/2, p-Erk1/2, p38, p-p38, JNK, p-JNK and LC3 (all 1:1000; Cell Signalling, Frankfurt, Germany). After an overnight incubation with the primary antibody at 4 °C and three washing steps with PBS/0.1% Tween 20 the membranes were incubated with peroxidase conjugated secondary antibody (1:5000, DIANOVA, Hamburg, Germany) for 1 h at room temperature. After a final incubation for 5 min with the Clarity chemoluminescence substrate (Bio-Rad Laboratories, München, Germany) signals were detected by densitometry using Bio-1D software version 15.01 (Vilber Lourmat). Actin signal intensities were used for normalization.

### 3.2 RNA extraction, cDNA synthesis and RT-qPCR

The PureLink RNA Mini Kit (Invitrogen, Darmstadt, Germany) was used to extract total RNA. The concentrations and the quality of the extracted RNAs were analyzed using a NanoDrop ND-1000 spectrophotometer (Peqlab, Erlangen, Germany). Synthesis of cDNA was done using 250 ng of extracted total RNA, 1 μl reverse transcriptase (Omniscript) (Qiagen, Hilden, Germany), 10 μM oligo-dT primer, 5 mM dNTPs and 10 U RNaseOut (Invitrogen, Karlsruhe, Germany). Twenty μl of this mixture were incubated for 1.5 h at 37 °C before cDNA was further diluted 1:5 with 10 mM Tricine (Carl Roth, Karlsruhe, Germany). Two μl of cDNA was then subjected to RT-qPCR analysis using primaQuant CYBR QPCR master mix (Steinbrenner Laborsysteme, Wiesenbach, Germany). The following program was run on a LineGene 9600 thermal cycler (Bior Technologies, Hangzhou, China): Preheating at 95 °C for 15 min and 40 cycles of denaturation at 95 °C for 15 s, annealing at 58 °C for 20 s and extension at 72 °C for 30 s followed by a melting curve analysis. The expression of *RPS13* (ribosomal protein S13) in the corresponding sample was used for normalization. All primers used are listed in the Supplemental Table 1.

### 3.3 Gene expression profiling

RNA from three GCTSC cell lines co-cultured with 45S5-BG (0.5 mg/ml) for 0, 12 and 48 h was subjected to gene expression profiling at the Genomics and Proteomics core facility of the Deutsche Krebsforschungszentrum (DKFZ) using an Affymetrix Human Clariom S Assay (Affymetrix/Thermo Fisher Scientific) and an iScan array scanner. After removal of outliers according to 2.5 Hampel's method, bead level data were quantile normalized. A student's *t*-test was used to identify significances between two experimental groups of log2 scaled expression levels.

### 3.4 Gene ontology enrichment analysis

Genome wide gene expression analysis and subsequent RT-qPCR validation revealed 25 genes that were upregulated >2.5-fold in GCTSC within 48h of treatment with 45S5-BG. These genes were submitted to a gene ontology enrichment analysis using ShinyGO (http://ge-lab.org/go/). This web-based enrichment analysis can link the input gene list with underlying biological processes and molecular functions. The results were ranked according to the false discovery rate (FDR) that represents the estimated probability that a gene set with a given enrichment score represents a false positive finding.

### 3.5 Immunofluorescence staining

GCTSC were cultured in 8-well chamber slides (Thermo Fisher) at a density of 50.000 cells per well. After treatment cells were fixed in ice-cold 100% methanol for 15 min at −20 °C, washed three times in PBS and blocked with PBS supplemented with 5% bovine serum albumin (BSA) and 0.3% Triton X-100 (both Thermo Fisher) for 60 min at room temperature. Blocking solution was replaced by the primary LC3 II antibody (Cell Signalling) diluted 1:200 in antibody dilution buffer consisting of PBS supplemented with 1% BSA and 0.3% Triton X-100. After incubation overnight at 4 °C cells were washed three times in PBS and incubated for 2 h at room temperature with an AlexaFluor-488 conjugated anti-mouse secondary antibody (Cell signalling) diluted 1:1000 in antibody dilution buffer. After three final PBS washes slides were mounted with RotiMount FluorCare mounting media containing DAPI as counterstain (Carl Roth, Germany) and photographed using a Keyence BZ-X810 microscope (Keyence, Neu-Isenburg, Germany).

### 3.6 Statistics

Descriptive statistics such as means, standard deviations and medians were calculated using SPSS software (Version 25; IBM, Armonk, NY, USA). The Mann-Whitney-U test was used to compare the experimental groups with p-values < 0.05 regarded as statistically significant.

## 4 Results

**Fig. 1:**
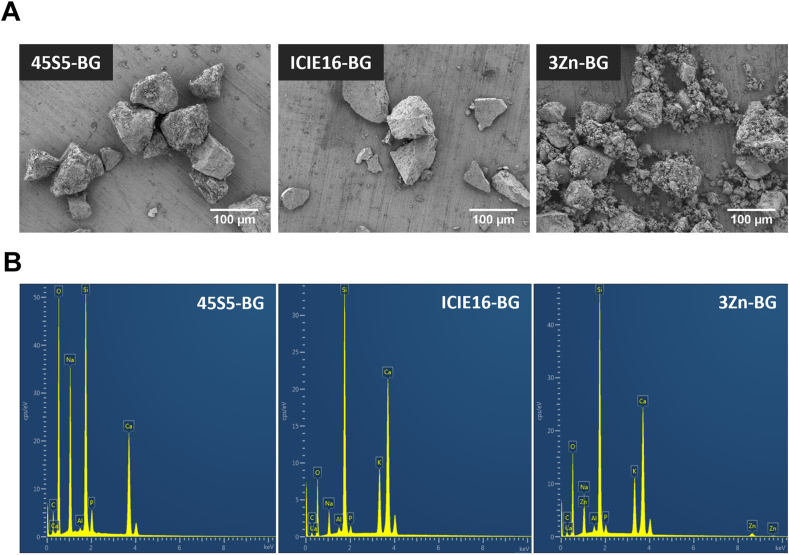
Characterization of bioactive glasses. A) SEM micrographs of the bioactive glass particles. B) EDX spectra of BG particles showing the elemental composition.

The polyhedral morphology of the sintered BG particles is shown in Fig. 1A. The estimated average particle size of the 45S5-BG, ICIE16-BG and 3Zn-BG granules was 65 ± 20, 62 ± 27 and 43 ± 19 μm, respectively. Fig. 1B depicts the elemental composition of the BG particles. The presence of distinctive elements of the BG compositions was detected, and negligible peaks related to aluminum in all samples, which could be attributed to the use of aluminium tape for the SEM/EDX sample preparation or contamination coming from the ceramic balls used during the milling process.

The analyses of the molecular mechanisms that mediate BG induced cytotoxicity were done using primary neoplastic stromal cell lines (GCTSC) isolated from GCTB tumor tissue. As control cells we used bone marrow derived stromal cells (BMSC) that were isolated from patients who underwent primary hip arthroplasty. Using these cell lines, we initially confirmed our previous findings according to which BGs exhibit a selective cytotoxicity towards GCTSC. Besides 45S5-BG which is already approved as a bone substitute material in clinical orthopedics, we included ICIE16-BG that is characterized by reduced sodium- but increased calcium content and 3Zn-BG, a modification of ICIE16-BG, supplemented with 3 mol % ZnO in exchange for CaO (Table 1). The selection of these BGs was based on the analysis of five different BGs in a previous study comparing the established 45S5-BG and ICIE16-BG with three newly developed BG-compositions containing the therapeutically active ions zinc (3Zn-BG), magnesium (3 Mg-BG), and boron (3B-BG) that have already been shown to influence and enhance the biological properties of the BGs. These ions were added in equal molarity compared to the ICIE16-BG composition by a partial replacement of the calcium ions. While 45S5-BG showed the most pronounced cytotoxic effect, 3Zn-BG showed the strongest osteogenic effect and ICIE16 an intermediate behaviour.

**Fig. 2:**
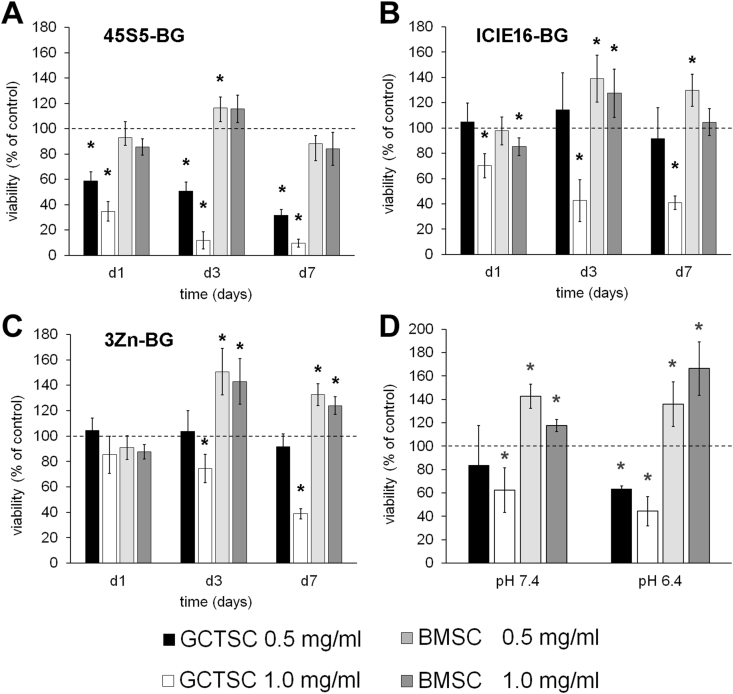
Selective cytotoxicity of bioactive glasses. GCTSC (n = 3) and BMSC (n = 3) cell lines were incubated with A) 45S5-BG, B) ICIE16-BG and C) 3Zn-BG at the indicated concentrations for 1, 3 and 7 days. Cell viability was quantified by WST-1 assay and expressed as percent of untreated control cells (dashed line). D) Cell viability of GCTSC (n = 4) and BMSC (n = 4) treated with 45S5-BG in medium with pH-values of 7.4 and 6.4, respectively. All experiments were done in triplicates (*p < 0.05 compared to untreated control).

As already shown for other GCTB cell lines in a previous study also the viability of the newly isolated GCTSCs was significantly decreased in a time- and concentration dependent manner after BG treatment [29]. The strongest effects were induced by 45S5-BG that was already visible after 24 h of treatment. In contrast, the viability of BMSC was not altered or even increased upon BG treatment (Fig. 2A–C). Since the tumor microenvironment is known to be acidic, with pH-values ranging from 6.4 to 7.0 [30], we additionally investigated whether the observed BG induced cytotoxicity is influenced by the pH-value of the culture medium. Four additional GCTSC and BMSC cell lines were treated with 45S5-BG in medium with normal (7.4) or acidic (6.4) pH-values for 3 days before cell viability was analyzed by WST-1 assay. The observed effects of 45S5-BG on the viability of GCTSC and BMSC were comparable and independent from the pH-value, suggesting that BG induced cytotoxicity is not inhibited by acidic tumor microenvironment (Fig. 2D).

**Fig. 3:**
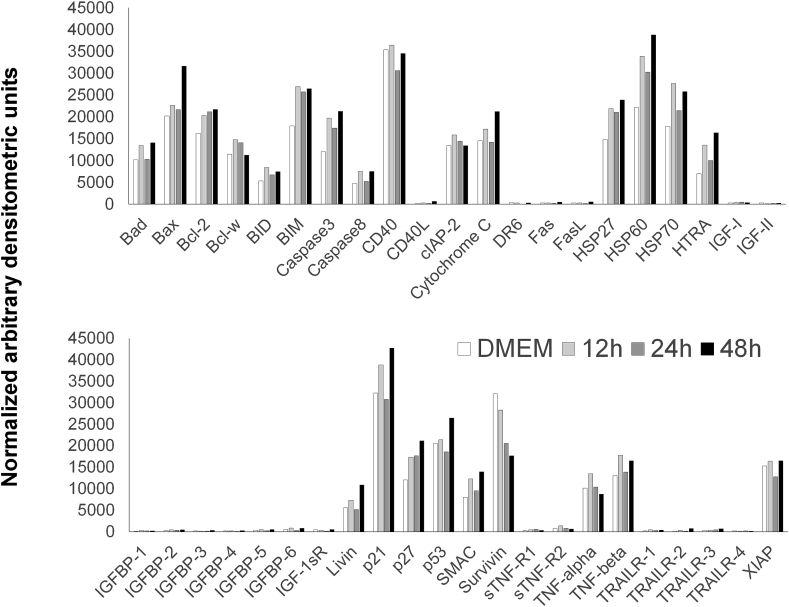
Antibody array analysis of GCTSC incubated with 45S5-BG for 12, 24 and 48 h. Signal intensities of 43 apoptotic factors were quantified by densitometry, background subtracted and normalized using positive control spots with a controlled amount of biotinylated antibody.

As the extent of the observed cytotoxicity was most evident using 45S5-BG, all further experiments were conducted using this BG. To verify whether apoptotic mechanisms are involved in BG induced cell death we performed an antibody array covering 43 apoptosis related proteins including cell surface receptors and their ligands, regulating factors from the BCL2 family, caspases, IGF family members, their binding proteins and several apoptosis regulating factors. Unexpectedly, none of these proteins was regulated >2-fold after 12, 24 or 48 h of BG treatment (Fig. 3 and Supplemental Fig. 1).

**Fig. 4:**
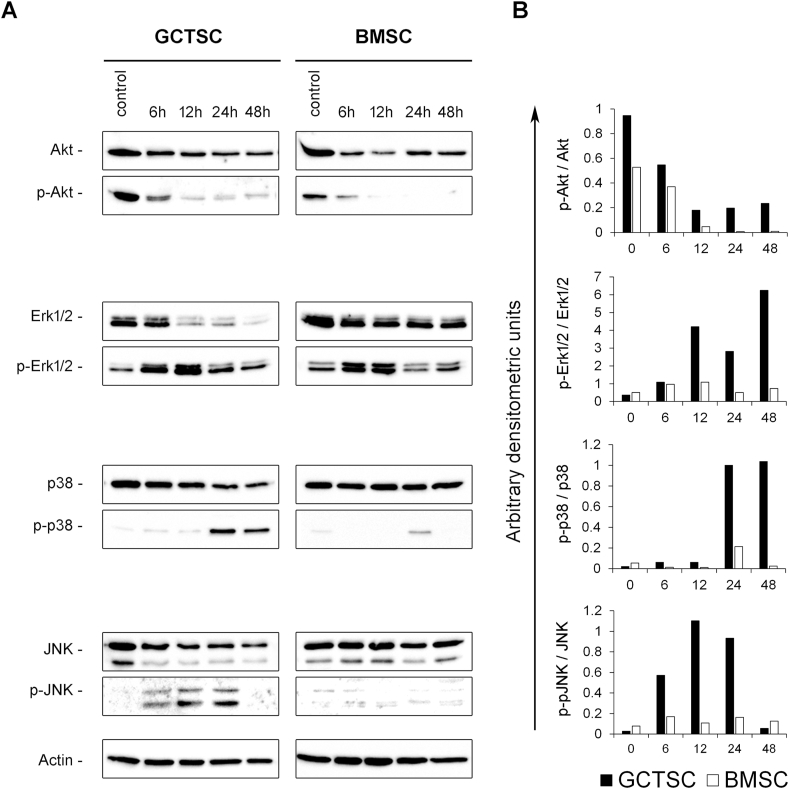
Activation of MAPK p38 and JNK in response to BG treatment. A) Western blot analyses of total and phosphorylated Akt, Erk1/2, p38 and JNK protein levels in GCTSC and BMSC after treatment with 45S5-BG (0.5 mg/ml). Actin protein levels served as loading control. B) Densitometric quantification of signal intensities normalized to the actin protein levels in the corresponding sample. The ratios of phosphorylated versus total protein levels are shown.

These data suggest, that BG treatment does not trigger the induction of apoptosis. We next analyzed the impact of BG treatment on the activation of Akt (protein kinase B) and MAPK (Mitogen-activated protein kinase) signalling pathways that are known to translate extracellular signals to cellular responses, thus controlling cell growth, differentiation and cell death. Activation of these pathways was monitored by the detection of total and phosphorylated protein levels using western-blot analyses. While treatment with 45S5-BG did not significantly affect total Akt protein we observed a rapid loss of pAkt, the phosphorylated active form of Akt in both GCTSC and BMSC. A comparable response of GCTSC and BMSC could also be observed concerning the activation of Erk1/2. Although total Erk1/2 levels declined to nearly undetectable levels within 48 h in GCTSC while they remained almost unchanged in BMSC, levels of phosphorylated Erk1/2 increased equally in both cell types rapidly within the first 6–12 h followed by a drop to control levels. In contrast to Akt and Erk1/2, we observed considerably different responses of GCTSC and BMSC concerning p38 and JNK (Jun N-terminal kinase) activation. A strong increase of p38 phosphorylation was induced after 24 h of BG treatment in GCTSC, while BMSC showed only weak p38 activation. JNK activation occurred much faster and was detectable already after 6 h of BG treatment in GCTSC while it was nearly undetectable in BMSC (Fig. 4A and B).

**Fig. 5:**
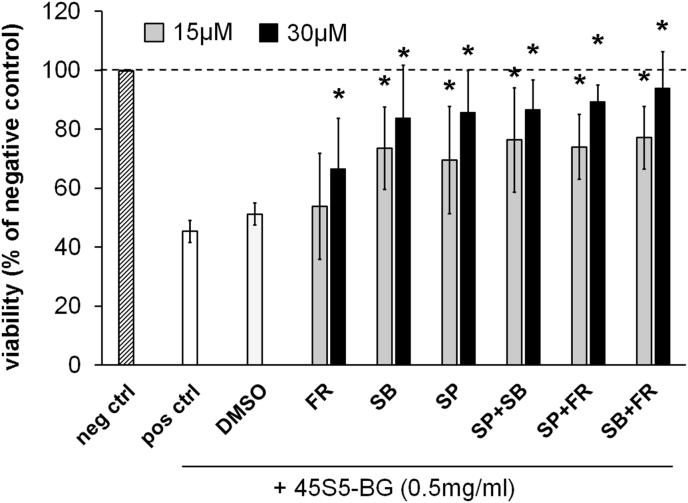
MAPK inhibitors counteract the BG induced cytotoxicity. GCTSC (n = 3) were treated with 45S5-BG (0.5 mg/ml) for 24 h with or without the addition of specific MAPK inhibitors. Cell viability was quantified by WST-1 assay and expressed as percent viability of untreated negative control cells (dashed line). (neg ctrl = untreated negative control cells; pos ctrl = positive control cell treated with 45S5-BG without the addition of MAPK inhibitors; DMSO = cells treated with the solvent DMSO; FR = Erk1/2 inhibitor FR180204; SB = p38 inhibitor SB202190; SP = JNK inhibitor SP600125) (*p < 0.05 compared to 45S5-BG treated cells without MAPK inhibitors).

To further investigate the importance of MAPKs for BG induced cytotoxicity we treated GCTSC with 45S5-BG in combination with specific MAPK inhibitors. While preincubation of the cells with the Erk1/2 inhibitor FR180204 showed only a minor effect on cell viability, the p38 inhibitor SB202190 and the JNK inhibitor SP600125 significantly counteracted BG induced cytotoxicity resulting in increased cell viabilities (84% and 86%) compared to cells treated without inhibitors (45%). Interestingly, combinations of these inhibitors did not show any additive effects (Fig. 5).

**Fig. 6:**
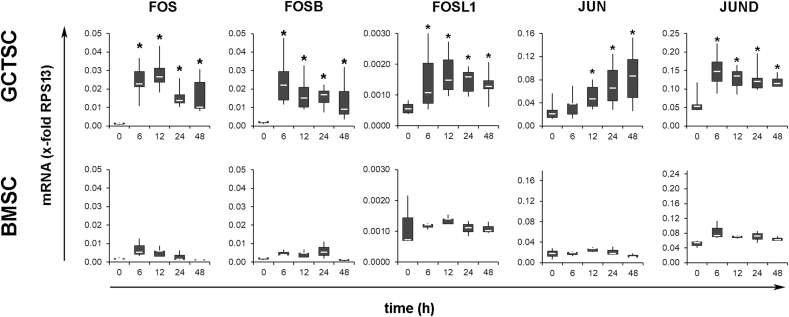
BG treatment increases the expression of transcription factors. RT-qPCR analysis of GCTSC (n = 6) and BMSC (n = 3) after incubation with 45S5-BG for the indicated times. Expression was normalized to the expression of the reference gene RPS13 in the corresponding sample. The white lines indicate the medians, the lower boundary of the box the 25th percentile and the upper box the 75th percentile. The whiskers indicate the highest and lowest values (*p < 0.05 compared to untreated control cells).

Because MAPKs are known to exert their effects mainly via triggering downstream transcription factors, we analyzed the expression of several members of the activator protein-1 (AP-1) transcription factor family by quantitative RT-qPCR. GCTSC responded to 45S5-BG treatment with a rapid induction of *FOS* (20-fold), *FOSB* (10.1-fold), *FOSL1* (2-fold) and *JUND* (2.8-fold) already after 6h. At later time points the expression of *JUN* increased up to 4.3-fold. Although mean *FOS* expression slightly increased after 6 h of treatment also in BMSC none of the analyzed transcription factors showed a significant upregulation in BMSC (Fig. 6).

**Fig. 7:**
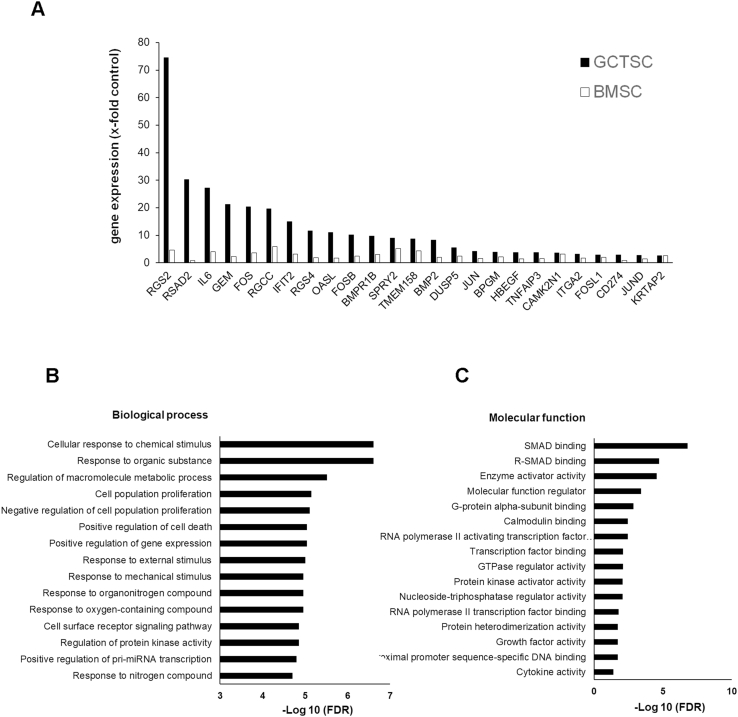
Comparison of gene expression profiles and enrichment analysis of differentially expressed genes. A) RT-qPCR analysis of differentially expressed genes. GCTSC (n = 6) and BMSC (n = 3) were treated with 45S5-BG (0.5 mg/ml) for 6, 12, 24 and 48 h. Median gene expression was calculated and genes with a >2.5-fold increase compared to untreated cells are shown. The values represent the highest increase of gene expression within the observed time period. B) Gene enrichment analysis of differentially expressed genes. Genes with an increased expression >2.5-fold after BG treatment and a >2.5-fold induction in GCTSC compared to BMSC were subjected to a gene ontology enrichment analysis using ShinyGO v0.61. The most significant biological processes (B) and molecular functions (C) are shown. The p-values are corrected for multiple testing using false discovery rate (FDR).

In order to identify further genes upregulated by 45S5-BG, including possible target genes of the upregulated transcription factors, we performed gene expression profiling of GCTSC (n = 3) treated with 45S5-BG for 12 and 48 h using an Affymetrix Human Clariom S Assay (Affymetrix/Thermo Fisher Scientific) at the Genomics and Proteomics core facility of the DKFZ. Array analysis confirmed the upregulation of the five transcription factors already identified by RT-qPCR and revealed further 20 genes that were upregulated >2.5-fold within 48h of BG treatment in all three GCTSC analyzed. Another five genes that were strongly upregulated in two out of three GCTSC were also included in the following RT-qPCR validation analysis. To validate the array data and to investigate the time course of gene expression, GCTSC (n = 6) and BMSC (n = 3) were treated with 45S5-BG for 6, 12, 24 and 48 h before gene expression was analyzed by RT-qPCR. In GCTSC a >2.5-fold induction by 45S5-BG treatment could be confirmed for 25 out of the 30 analyzed genes (Fig. 7A).

**Fig. 8:**
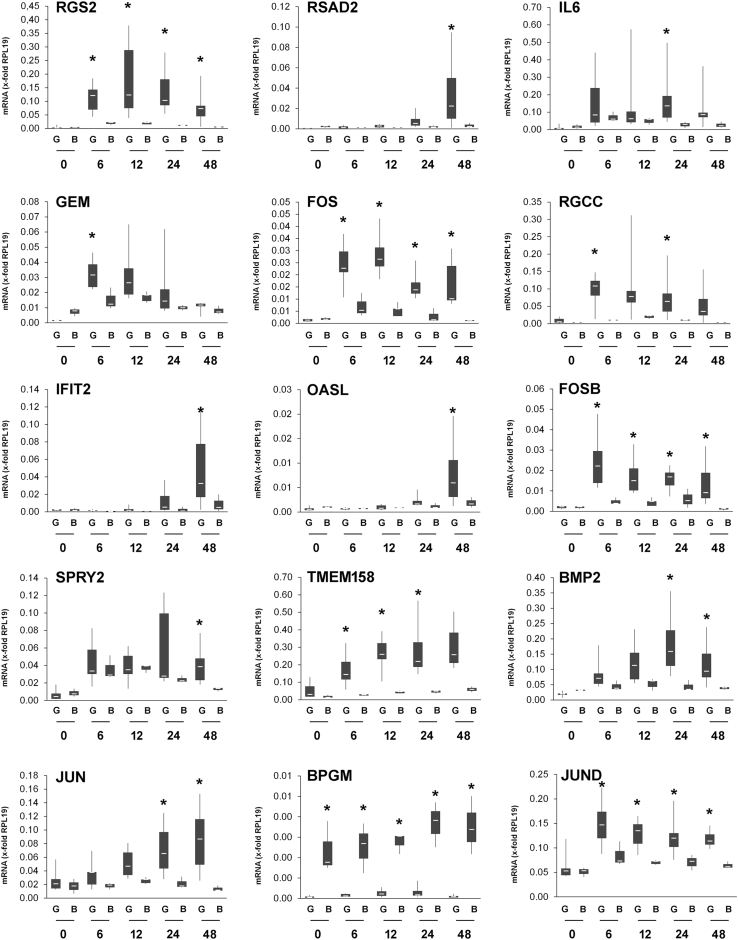
Validation of 45S5-BG induced gene expression. GCTSC (G) (n = 6) and BMSC (B) (n = 3) were co-cultured with 45S5-BG (0.5 mg/ml) for the indicated times before gene expression was analyzed by RT-qPCR. Gene expression was normalized to the expression of the reference gene RPS13 in the corresponding sample. Genes with significant higher expression in GCTSC compared to BMSC at least at one time point are shown. The white lines indicate the medians, the lower boundary of the box the 25th percentile and the upper box the 75th percentile. The whiskers indicate the highest and lowest values (*p < 0.05 compared to untreated control cells).

These genes were subjected to a gene ontology enrichment analysis using ShinyGo v0.61 (http://ge-lab.org/go/). The upregulated genes are significantly enriched in the following biological processes: response to chemical, organic, and mechanical stimuli, negative regulation of proliferation, positive regulation of gene expression and cell death, cell surface receptor signalling and more. Amongst the enriched molecular functions, we identified SMAD binding, G-protein and calmodulin binding, transcription factor binding, protein kinase activator activity, GTPase activation and others (Fig. 7B and C). While some of the identified genes were upregulated in GCTSC and BMSC to a comparable extend, a subset of 15 genes was exclusively induced in GCTSC or induced significantly stronger (>2.0-fold) in GCTSC compared to BMSC, suggesting a possible role in selective BG induced cytotoxicity. Interestingly, most of these genes can be grouped into genes involved in G-protein signalling, tumor suppressors and interferon-induced genes (Fig. 8).

**Fig. 9:**
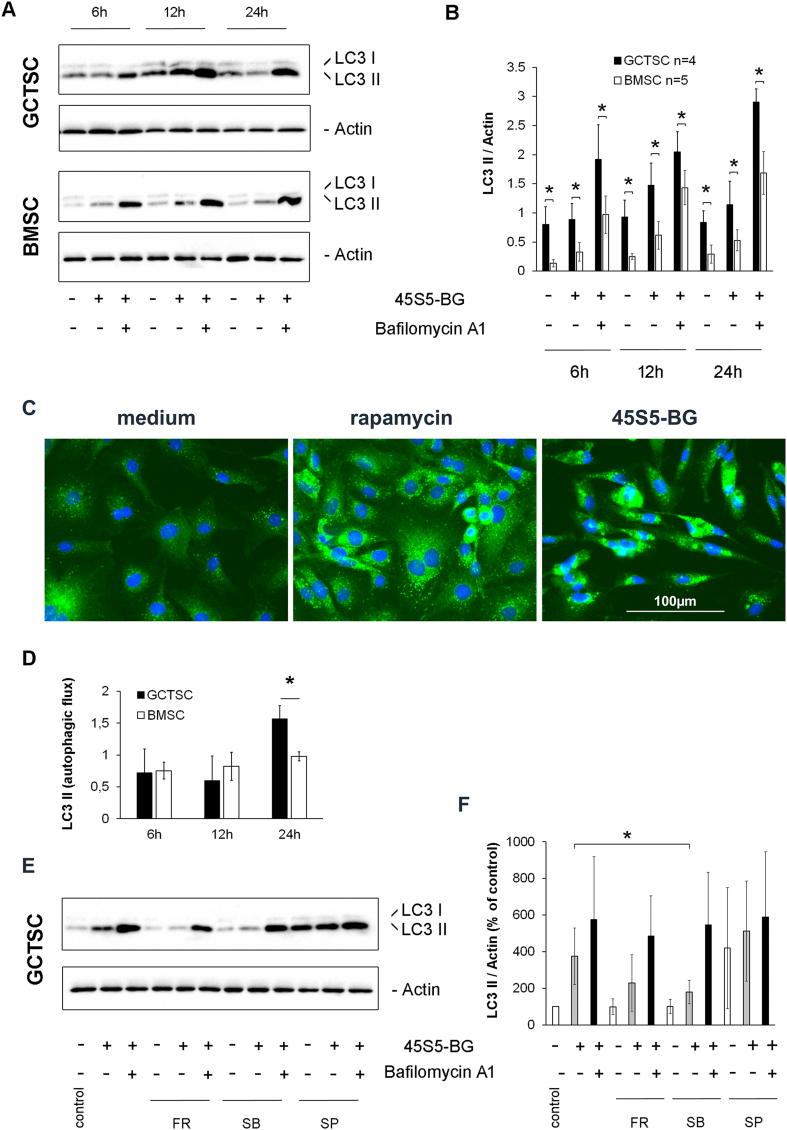
Induction of autophagy by BG treatment. A) Western blot analysis of LC3 levels in GCTSC and BMSC treated with 45S5-BG (0.5 mg/ml) with or without the addition of Bafilomycin A1 (20 nM). Actin protein levels were quantified as loading control. Representative western blots are shown. B) Densitometric quantification of LC3 II signals shown in A). C) Immunofluorescence staining of LC3 II in GCTSC treated with rapamycin (1 μM) or 45S5-BG (1 mg/ml) for 24h. Representative photographs are shown. D) Calculated autophagic flux in GCTSC and BMSC treated with 45S5-BG. E) Inhibition of autophagy by MAPK inhibitors. GCTSC (n = 3) were treated for 12 h with 45S5-BG (0.5 mg/ml) with or without the addition of Bafilomycin A1 (20 nM) in combination with the ERK1/2 inhibitor FR180204 (FR) the p38 inhibitor SB202190 (SB) and the JNK inhibitor SP600125 (SP), respectively (30 μM each). F) Densitometric quantification of LC3 II signals shown in D) (*p < 0.05).

Since we could not detect any signs of apoptosis in response to BG treatment, we assumed a possible role of autophagy. A reliable method to monitor autophagy is the detection of the microtubule-associated protein 1A/1B-light chain 3 (LC3). During autophagy, autophagosomes transfer cytosolic proteins to lysosomes where they are degraded. During this process the cytosolic form of LC3 (LC3 I) is converted to LC3 II by conjugation to phosphatidylethanolamine. LC3 II is then incorporated into autophagosomal membranes and can thus be used to quantify the formation of autophagosomes. In addition to the steady state levels of LC3 II at a given time point, the autophagic flux can be determined by comparing LC3 II levels with and without the addition of lysosomal protease inhibitors like bafilomycin A1 that blocks degradation of LC3 II during autophagy. Using this approach, we detected a strong increase of LC3 II levels after 12 h of BG treatment that were further enhanced in the presence of bafilomycin A1, indicating an increase of autophagosomes in response to BG. In both, untreated cells and BG-treated cells, the detected LC3 II levels were significantly higher in GCTSC compared to BMSC (Fig. 9A and B). Visualization of LC3 II positive autophagosomes in GCTSC was done using immunofluorescence staining of untreated cells or cells treated with the autophagy inducer rapamycin and 45S5-BG, respectively. While control cells only contained a few autophagosomes, rapamycin and 45S5-BG treated cells showed a massive increase of LC3 II positive autophagosomes. In addition, 45S5-BG treated cells showed morphologic changes to a more slender and elongated phenotype already after 24h of treatment (Fig. 9C).

Considering the autophagic flux represented by the difference of LC3 II levels after 45S5-BG treatment with and without the addition of bafilomycin A1, a significant difference between GCTSC and BMSC could be seen after 24 h of treatment (Fig. 9D). Whether BG induced autophagy depends on MAPK activity was analyzed by the addition of specific MAPK inhibitors. After 12 h of BG treatment the Erk1/2 inhibitor FR180204 and the p38 inhibitor SB202190 markedly inhibited the increase of LC3 II levels seen without inhibitors, indicating a crucial role of MAPKs in the BG induced autophagy. In contrast to Erk1/2 and p38 inhibition, the JNK inhibitor SP600125 even increased autophagy levels (Fig. 9 E and F).

## 5 Discussion

In clinical routine, GCTB is a challenging tumor with unpredictable behavior, destructive growth and very high recurrence rates. We hypothesized that BGs might be a promising and new therapeutic approach due to our observation of a cytotoxic effect against tumor cells and their known osteogenic properties that could be exploited for the actual repair of bone defects generated by tumor growth and surgery. However, the molecular mechanisms that mediate BG induced cytotoxicity, especially GCTSC, are completely unknown. Therefore, the aim of this study was the investigation of these mechanisms.

We observed a rapid and significant decrease of cell viability in response to BG treatment, exclusively in tumor cells that was unexpectedly not associated with any signs of apoptosis. However, we observed activation of mitogen-activated protein kinases (MAPK), especially p38 and JNK also called stress activated protein kinases due to their known activation by environmental and genotoxic stress. MAPKs play important roles in the regulation of proliferation, differentiation, cell cycle progression, senescence and cell death. Interestingly, both, p38 and JNK have also been shown to control the balance of apoptosis and autophagy [31] and to regulate induction of autophagy in response to a variety of stimuli [[32], [33], [34], [35]]. In our study, BG induced MAPK activation was considerably stronger in GCTSC compared to BMSC which could be an indication of the observed different sensitivities of these cell types towards BG treatment. In addition, the strong activation of Erk1/2 but low activation od p38 observed in BMSC confirm the observation of other groups showing that BGs induce osteogenic differentiation mainly through activation of Erk1/2 rather than p38 [36]. A major target of p38 and JNK is the transcription factor AP-1 (activator protein 1) that is composed of Fos and Jun family members [37]. Both, JNK and p38 have been shown to activate these transcription factors, thus controlling the expression of numerous target genes including genes associated with cell death [38]. The inhibition of BG induced cell death by MAPK inhibitors observed in our study as well as the rapid induction of AP-1 family members in response to BG treatment indicate a central role of this signaling axis for BG induced cytotoxicity. Beside the rapid upregulation of transcription factors belonging to the AP-1 family we identified 11 other genes that were induced in response to BG treatment exclusively or significantly stronger in GCTSC compared to BMSC, suggesting a crucial role in the observed tumor or cell specific cytotoxicity. Most of these genes can be assigned to one of the functional groups; genes involved in G-protein signalling, tumor suppressors and interferon-induced genes.

Among the G-protein signalling genes *RGS2* (regulator of G-protein signalling), a negative regulator of G-protein coupled receptors, showed the strongest induction. *RGS2* overexpression has been shown to inhibit proliferation in MCF7 breast cancer cells and HEK293T cells [39]. Further, downregulation has been observed in several types of cancer including colorectal cancer [40] and prostate cancer [41] suggesting a tumor suppressor function. Interestingly, RGS2 has also been shown to promote translation of ATF4 (Activating Transcription Factor 4) [42] that in turn is essential for stress-induced autophagy gene expression [43]. The Ras like GTP-binding protein *GEM* belongs to the RAD/GEM family and is associated with the inner face of the plasma membrane and regulates, like Ras, receptor-mediated signal transduction and might thus be involved in the observed BG induced MAPK activation. Tumor suppressor properties have also been observed for *RGCC* (regulator of cell cycle) also known as RGC-32 (response gene to complement) that is involved in the regulation of cell cycle progression. In colon cancer cells *RGCC* knockdown affected the expression of genes involved in chromatin assembly and increased the number of cells within the S- and G2/M-phase, suggesting a role in the regulation of chromatin assembly [44]. *SPRY2* (Sprouty homolog 2) belongs to a family of negative regulators of receptor tyrosine kinase signalling that has been shown to regulate pathways involved in proliferation and cell death in human embryonic stem cells [45]. MicroRNA mediated downregulation of SPRY2 has been observed in several cancers affirming its tumor suppressor properties [[46], [47], [48]]. TMEM158 (transmembrane protein 158) also known as RIS1 (Ras-induced senescence 1) was identified in Ras senescent human fibroblasts [49] and is upregulated upon Ras activation. The Ras oncogene encodes a small GTP-binding protein that transduce mitogenic signals and is frequently constitutively activated in human cancers. However, for the activation of proliferation and tumorigenesis Ras must cooperate with certain other oncogenic alterations like overexpression of c-Myc or loss of p53. It has been shown that without these additional events Ras acts as tumor suppressor leading to cell cycle arrest and cellular senescence [50]. Thus, our observation of a strong upregulation of TMEM158 in combination with the activation of the JNK and p38 MAPK signalling pathways in response to BG treatment might reflect this anti-proliferative Ras effect.

In contrast to the rapid induction of transcription factors and tumor suppressors within the first 6h of BG treatment, we detected an increased expression of several interferon-induced genes at later time points (48h). One of these genes is *RSAD2* (Radical S-Adenosyl Methionine Domain Containing 2) that is involved in viral defence mechanisms, for example through inhibition of DNA and RNA virus replication [51]. Although *RSAD2* expression was strongly induced after 48h of 45S5-BG treatment we could not observe increased expression of interferon genes within our array analysis. However, interferon induced genes like *RSAD2* and *IFIT2* (interferon induced protein with tetratricopeptide repeats 2) that were also strongly upregulated in response to BG treatment can also be induced by several other signalling pathways including Toll-like receptor 3, virus infection or pathogen-associated molecular patterns (PAMPs) [52]. *IFIT2* has been reported to be involved in the regulation of cell cycle, apoptosis, tumor colonization, and viral replication [53,54]. The observation of decreased *IFIT2* expressions in lung and gastric cancer that is associated with tumor progression and poor survival supports its tumor suppressor function [55,56]. Finally, *OASL* (oligoadenylate synthase-like protein) another interferon induced gene upregulated by BG treatment is a key antiviral factor that regulates early phase of viral infection by degrading viral RNA [57]. The strong induction of these interferon-induced genes in BG treated GCTSC suggests that BGs trigger cellular responses comparable to those seen after viral or bacterial infections. Although array analysis demonstrated that none of the interferon genes is affected by BG treatment, upregulation of interferon-induced genes seems to play an important role in BG induced cell death.

BG induced genes that do not fit in the categories described above but have already been associated with autophagy are *IL6* (interleukin 6) and *BMP2* (bone morphogenetic protein 2). Induction of IL-6 secretion has been shown in HeLa cells upon starvation induced autophagy. When these cells were combined as bystander cells with irradiated HeLa cells, irradiation induced autophagy was significantly reduced, suggesting that autophagy induced IL6 production functions as a rescue signal sent to surrounding cells [58]. BMP2 belongs to the TGF-ß superfamily and plays an important role in bone and cartilage formation and many processes in early development including regulation of cell growth, apoptosis and differentiation. Beside the known signalling through intracellular Smad proteins [59] the involvement of MAPKs, such as p38 and JNK, has also been observed during BMP-2 signalling [60] and cells overexpressing BMP-2 have been shown to overexpress autophagy related factors [61].

A possible mechanism by which induction of the above-described genes might mediate BG induced cytotoxicity is the activation of autophagy. Although autophagy usually is a self-degradative process with cell-protective properties it has been shown that hyperactivation of autophagy can function as cell suicide mechanism that is implicated in tumorigenesis and the effectiveness of cancer chemotherapeutics [62]. In our study we observed increased LC3 II levels in response to 45S5-BG treatment demonstrating the induction of autophagy that was significantly more pronounced in GCTSC compared to BMSC. However, since we observed autophagy in both cell types, we cannot conclude from our data that the observed selective death of tumor cells is solely triggered by autophagy.

In summary, our data demonstrate that BG treatment leads to rapid activation of the MAPKs p38 and JNK and, as a result, increased expression of AP-1 transcription factors and a small group of target genes in GCTSC but not in BMSC. The observed time course of gene expression and the fact that BG induced autophagy peaks after 12 h of treatment suggests that upregulation of genes involved in G-protein signalling like *RGS2* and *GEM*, as well as tumor suppressors like *RGCC*, *SPRY2* and *TMEM158* represent the early response to BG treatment, probably involved in the induction of autophagic cell death. At later time points several interferon-induced genes are upregulated when cell death is already triggered, suggesting a rescue mechanism through unspecific induction of antiviral defence programs.

However, the precise role of the identified genes in BG induced cytotoxicity as well as the exact mechanism of MAPK activation still have to be addressed. In addition, we yet do not know how the different glass compositions contribute to the different degrees of cytotoxicity. Notably, the induction of tumor cell specific cytotoxicity is not possible using BG conditioned media, media with altered pH-value or by transwell experiments with BGs and cells separated by a membrane as shown in our previous study. These data indicate that the cytotoxic effect of BGs is independent from the BG induced pH-shift and dissolution products, but depends on a direct contact of cells and BGs. Our data further suggest, that the acidic microenvironment does not inhibit BG induced cytotoxicity. One could therefore assume that the physical parameters like BG surface properties and particle size rather than the chemical parameters play an important role in the cytotoxic effects of BGs. However, the particle size of 45S5-BG and ICIE16-BG used in this study is quite similar although cytotoxicity is different, indicating that particle size alone is not sufficient to determine BG mediated cytotoxicity. In addition, the observed induction of several interferon-induced genes might be a consequence of a direct activation of cell surface receptors including Toll-like receptors (TLRs) that are known to act as cell surface sensors that lead upon activation to the induction of immune and inflammatory genes. Although TLRs mainly function as detectors of pathogens, also interactions of TLRs with biomaterials have already been shown to induce the expression pro-inflammatory cytokines [63]. These possible mechanisms and especially the effectiveness of BG treatment under in vivo conditions have to be investigated in future studies. So far, our data support and complement our previous findings and demonstrate that BGs might be promising biomaterials suitable for the development of new therapeutic approaches for GCTB treatment combining the reduction of tumor recurrences by elimination of tumor cells with enhanced BMSC mediated bone repair.

## Conflict of interest

The authors have no conflict of interest to declare.

## CRediT authorship contribution statement

**Joerg Fellenberg:** Conceptualization, Methodology, Validation, Formal analysis, Investigation, Visualization, Supervision, Writing – original draft, Writing – review & editing, Project administration, Funding acquisition. **Sarina Losch:** Investigation, Validation. **Burkhard Lehner:** Conceptualization, Resources. **Marcela Arango-Ospina:** Investigation, Validation, Writing – review & editing. **Aldo R. Boccaccini:** Conceptualization, Resources, Writing – review & editing, Funding acquisition, Project administration. **Fabian Westhauser:** Conceptualization, Formal analysis, Writing – review & editing, Project administration, Funding acquisition.

## Declaration of competing interest

The authors declare that there are no conflicts of interest.

## References

[1] Verschoor A.J., Bovee J., Mastboom M.J.L., Sander Dijkstra P.D., Van De Sande M.A.J., Gelderblom H. (2018). Incidence and demographics of giant cell tumor of bone in The Netherlands: first nationwide Pathology Registry Study. Acta Orthop..

[2] Noh B.J., Park Y.K. (2018). Giant cell tumor of bone: updated molecular pathogenesis and tumor biology. Hum. Pathol..

[3] Atkins G.J., Haynes D.R., Graves S.E., Evdokiou A., Hay S., Bouralexis S., Findlay D.M. (2000). Expression of osteoclast differentiation signals by stromal elements of giant cell tumors. J. Bone Miner. Res..

[4] Behjati S., Tarpey P.S., Presneau N., Scheipl S., Pillay N., Van Loo P., Wedge D.C., Cooke S.L., Gundem G., Davies H., Nik-Zainal S., Martin S., McLaren S., Goodie V., Robinson B., Butler A., Teague J.W., Halai D., Khatri B., Myklebost O., Baumhoer D., Jundt G., Hamoudi R., Tirabosco R., Amary M.F., Futreal P.A., Stratton M.R., Campbell P.J., Flanagan A.M. (2013). Distinct H3F3A and H3F3B driver mutations define chondroblastoma and giant cell tumor of bone. Nat. Genet..

[5] Lutsik P., Baude A., Mancarella D., Oz S., Kuhn A., Toth R., Hey J., Toprak U.H., Lim J., Nguyen V.H., Jiang C., Mayakonda A., Hartmann M., Rosemann F., Breuer K., Vonficht D., Grunschlager F., Lee S., Schuhmacher M.K., Kusevic D., Jauch A., Weichenhan D., Zustin J., Schlesner M., Haas S., Park J.H., Park Y.J., Oppermann U., Jeltsch A., Haller F., Fellenberg J., Lindroth A.M., Plass C. (2020). Globally altered epigenetic landscape and delayed osteogenic differentiation in H3.3-G34W-mutant giant cell tumor of bone. Nat. Commun..

[6] Balke M., Schremper L., Gebert C., Ahrens H., Streitbuerger A., Koehler G., Hardes J., Gosheger G. (2008). Giant cell tumor of bone: treatment and outcome of 214 cases. J. Cancer Res. Clin. Oncol..

[7] Omlor G.W., Lange J., Streit M., Gantz S., Merle C., Germann T., Mechtersheimer G., Fellenberg J., Lehner B. (2019). Retrospective analysis of 51 intralesionally treated cases with progressed giant cell tumor of the bone: local adjuvant use of hydrogen peroxide reduces the risk for tumor recurrence. World J. Surg. Oncol..

[8] Algawahmed H., Turcotte R., Farrokhyar F., Ghert M. (2010). High-speed burring with and without the use of surgical adjuvants in the intralesional management of giant cell tumor of bone: a systematic review and meta-analysis. Sarcoma.

[9] Balke M., Schremper L., Gebert C., Ahrens H., Streitbuerger A., Koehler G., Hardes J., Gosheger G. (2008). Giant cell tumor of bone: treatment and outcome of 214 cases. J. Cancer Res. Clin. Oncol..

[10] Trieb K., Bitzan P., Lang S., Dominkus M., Kotz R. (2001). Recurrence of curetted and bone-grafted giant-cell tumours with and without adjuvant phenol therapy. Eur. J. Surg. Oncol..

[11] Turcotte R.E., Wunder J.S., Isler M.H., Bell R.S., Schachar N., Masri B.A., Moreau G., Davis A.M., Canadian Sarcoma G. (2002). Giant cell tumor of long bone: a Canadian Sarcoma Group study. Clin. Orthop. Relat. Res..

[12] Errani C., Tsukamoto S., Mavrogenis A.F. (2017). How safe and effective is denosumab for bone giant cell tumour?. Int. Orthop..

[13] Urakawa H., Yonemoto T., Matsumoto S., Takagi T., Asanuma K., Watanuki M., Takemoto A., Naka N., Matsumoto Y., Kawai A., Kunisada T., Kubo T., Emori M., Hiraga H., Hatano H., Tsukushi S., Nishida Y., Akisue T., Morii T., Takahashi M., Nagano A., Yoshikawa H., Sato K., Kawano M., Hiraoka K., Tanaka K., Iwamoto Y., Ozaki T. (2018). Clinical outcome of primary giant cell tumor of bone after curettage with or without perioperative denosumab in Japan: from a questionnaire for JCOG 1610 study. World J. Surg. Oncol..

[14] Luengo-Alonso G., Mellado-Romero M., Shemesh S., Ramos-Pascua L., Pretell-Mazzini J. (2019). Denosumab treatment for giant-cell tumor of bone: a systematic review of the literature. Arch. Orthop. Trauma. Surg..

[15] Hench L.L. (2006). The story of Bioglass. J. Mater. Sci. Mater. Med..

[16] Baino F., Hamzehlou S., Kargozar S. (2018). Bioactive glasses: where are we and where are we going?. J. Funct. Biomater..

[17] Krishnan V., Lakshmi T. (2013). Bioglass: a novel biocompatible innovation. "J. Adv. Pharm. Technol. Research"" (JAPTR)".

[18] Rizwan M., Hamdi M., Basirun W.J. (2017). Bioglass(R) 45S5-based composites for bone tissue engineering and functional applications. J. Biomed. Mater. Res..

[19] Bosetti M., Cannas M. (2005). The effect of bioactive glasses on bone marrow stromal cells differentiation. Biomaterials.

[20] Kaufmann E.A., Ducheyne P., Shapiro I.M. (2000). Effect of varying physical properties of porous, surface modified bioactive glass 45S5 on osteoblast proliferation and maturation. J. Biomed. Mater. Res..

[21] Xynos I.D., Edgar A.J., Buttery L.D., Hench L.L., Polak J.M. (2000). Ionic products of bioactive glass dissolution increase proliferation of human osteoblasts and induce insulin-like growth factor II mRNA expression and protein synthesis. Biochem. Biophys. Res. Commun..

[22] Gorustovich A.A., Roether J.A., Boccaccini A.R. (2010). Effect of bioactive glasses on angiogenesis: a review of in vitro and in vivo evidences. Tissue Eng. B Rev..

[23] Rahaman M.N., Day D.E., Bal B.S., Fu Q., Jung S.B., Bonewald L.F., Tomsia A.P. (2011). Bioactive glass in tissue engineering. Acta Biomater..

[24] Jones J.R. (2015). Reprint of: review of bioactive glass: from Hench to hybrids. Acta Biomater..

[25] Ciraldo F.E., Boccardi E., Melli V., Westhauser F., Boccaccini A.R. (2018). Tackling bioactive glass excessive in vitro bioreactivity: preconditioning approaches for cell culture tests. Acta Biomater..

[26] Hohenbild F., Arango-Ospina M., Moghaddam A., Boccaccini A.R., Westhauser F. (2020). Preconditioning of bioactive glasses before introduction to static cell culture: what is really necessary?. Meth. prot..

[27] Brito A.F., Antunes B., Dos Santos F., Fernandes H.R., Ferreira J.M.F. (2017). Osteogenic capacity of alkali-free bioactive glasses. In vitro studies. J. Biomed. Mater. Res. B Appl. Biomater..

[28] Yan L., Li H., Xia W. (2020). Bioglass could increase cell membrane fluidity with ion products to develop its bioactivity. Cell Prolif.

[29] Westhauser F., Arango-Ospina M., Losch S., Wilkesmann S., Lehner B., Ali M.S., Peukert W., Boccaccini A.R., Fellenberg J. (2021). Selective and caspase-independent cytotoxicity of bioactive glasses towards giant cell tumor of bone derived neoplastic stromal cells but not to bone marrow derived stromal cells. Biomaterials.

[30] Boedtkjer E., Pedersen S.F. (2020). The acidic tumor microenvironment as a driver of cancer. Annu. Rev. Physiol..

[31] Sui X., Kong N., Ye L., Han W., Zhou J., Zhang Q., He C., Pan H. (2014). p38 and JNK MAPK pathways control the balance of apoptosis and autophagy in response to chemotherapeutic agents. Cancer Lett..

[32] Cui Q., Tashiro S., Onodera S., Minami M., Ikejima T. (2007). Oridonin induced autophagy in human cervical carcinoma HeLa cells through Ras, JNK, and P38 regulation. J. Pharmacol. Sci..

[33] Hu C., Zou M.J., Zhao L., Lu N., Sun Y.J., Gou S.H., Xi T., Guo Q.L. (2012). E Platinum, a newly synthesized platinum compound, induces autophagy via inhibiting phosphorylation of mTOR in gastric carcinoma BGC-823 cells. Toxicol. Lett..

[34] Sun T., Li D., Wang L., Xia L., Ma J., Guan Z., Feng G., Zhu X. (2011). c-Jun NH2-terminal kinase activation is essential for up-regulation of LC3 during ceramide-induced autophagy in human nasopharyngeal carcinoma cells. J. Transl. Med..

[35] Zhou F., Yang Y., Xing D. (2011). Bcl-2 and Bcl-xL play important roles in the crosstalk between autophagy and apoptosis. FEBS J..

[36] Ojansivu M., Hyvari L., Kellomaki M., Hupa L., Vanhatupa S., Miettinen S. (2018). Bioactive glass induced osteogenic differentiation of human adipose stem cells is dependent on cell attachment mechanism and mitogen-activated protein kinases. Eur. Cell. Mater..

[37] Eferl R., Wagner E.F. (2003). AP-1: a double-edged sword in tumorigenesis. Nat. Rev. Cancer.

[38] Shaulian E., Karin M. (2002). AP-1 as a regulator of cell life and death. Nat. Cell Biol..

[39] Lyu J.H., Park D.W., Huang B., Kang S.H., Lee S.J., Lee C., Bae Y.S., Lee J.G., Baek S.H. (2015). RGS2 suppresses breast cancer cell growth via a MCPIP1-dependent pathway. J. Cell. Biochem..

[40] Jiang Z., Wang Z., Xu Y., Wang B., Huang W., Cai S. (2010). Analysis of RGS2 expression and prognostic significance in stage II and III colorectal cancer. Biosci. Rep..

[41] Linder A., Hagberg Thulin M., Damber J.E., Welen K. (2018). Analysis of regulator of G-protein signalling 2 (RGS2) expression and function during prostate cancer progression. Sci. Rep..

[42] Wang C.J., Chidiac P. (2019). RGS2 promotes the translation of stress-associated proteins ATF4 and CHOP via its eIF2B-inhibitory domain. Cell. Signal..

[43] B'Chir W., Maurin A.C., Carraro V., Averous J., Jousse C., Muranishi Y., Parry L., Stepien G., Fafournoux P., Bruhat A. (2013). The eIF2alpha/ATF4 pathway is essential for stress-induced autophagy gene expression. Nucleic Acids Res..

[44] Vlaicu S.I., Tegla C.A., Cudrici C.D., Fosbrink M., Nguyen V., Azimzadeh P., Rus V., Chen H., Mircea P.A., Shamsuddin A., Rus H. (2010). Epigenetic modifications induced by RGC-32 in colon cancer. Exp. Mol. Pathol..

[45] Felfly H., Klein O.D. (2013). Sprouty genes regulate proliferation and survival of human embryonic stem cells. Sci. Rep..

[46] Li Y., Chen H., She P., Chen T., Chen L., Yuan J., Jiang B. (2018). microRNA-23a promotes cell growth and metastasis in gastric cancer via targeting SPRY2-mediated ERK signaling. Oncol. Lett..

[47] Xiao S., Yang M., Yang H., Chang R., Fang F., Yang L. (2018). miR-330-5p targets SPRY2 to promote hepatocellular carcinoma progression via MAPK/ERK signaling. Oncogenesis.

[48] He Y., Ge Y., Jiang M., Zhou J., Luo D., Fan H., Shi L., Lin L., Yang L. (2018). MiR-592 promotes gastric cancer proliferation, migration, and invasion through the PI3K/AKT and MAPK/ERK signaling pathways by targeting Spry2. Cell. Physiol. Biochem..

[49] Barradas M., Gonos E.S., Zebedee Z., Kolettas E., Petropoulou C., Delgado M.D., Leon J., Hara E., Serrano M. (2002). Identification of a candidate tumor-suppressor gene specifically activated during Ras-induced senescence. Exp. Cell Res..

[50] Serrano M., Lin A.W., McCurrach M.E., Beach D., Lowe S.W. (1997). Oncogenic ras provokes premature cell senescence associated with accumulation of p53 and p16INK4a. Cell.

[51] Zahoor M.A., Xue G., Sato H., Murakami T., Takeshima S.N., Aida Y. (2014). HIV-1 Vpr induces interferon-stimulated genes in human monocyte-derived macrophages. PLoS One.

[52] Diamond M.S., Farzan M. (2013). The broad-spectrum antiviral functions of IFIT and IFITM proteins. Nat. Rev. Immunol..

[53] Stawowczyk M., Van Scoy S., Kumar K.P., Reich N.C. (2011). The interferon stimulated gene 54 promotes apoptosis. J. Biol. Chem..

[54] Lai K.C., Liu C.J., Chang K.W., Lee T.C. (2013). Depleting IFIT2 mediates atypical PKC signaling to enhance the migration and metastatic activity of oral squamous cell carcinoma cells. Oncogene.

[55] Su W., Xiao W., Chen L., Zhou Q., Zheng X., Ju J., Jiang J., Wang Z. (2019). Decreased IFIT2 expression in human non-small-cell lung cancer tissues is associated with cancer progression and poor survival of the patients. OncoTargets Ther..

[56] Chen L., Zhai W., Zheng X., Xie Q., Zhou Q., Tao M., Zhu Y., Wu C., Jiang J. (2018). Decreased IFIT2 expression promotes gastric cancer progression and predicts poor prognosis of the patients. Cell. Physiol. Biochem..

[57] Choi U.Y., Kang J.S., Hwang Y.S., Kim Y.J. (2015). Oligoadenylate synthase-like (OASL) proteins: dual functions and associations with diseases. Exp. Mol. Med..

[58] Kong E.Y., Cheng S.H., Yu K.N. (2018). Induction of autophagy and interleukin 6 secretion in bystander cells: metabolic cooperation for radiation-induced rescue effect?. J. Radiat. Res..

[59] Shi Y., Massague J. (2003). Mechanisms of TGF-beta signaling from cell membrane to the nucleus. Cell.

[60] Guicheux J., Lemonnier J., Ghayor C., Suzuki A., Palmer G., Caverzasio J. (2003). Activation of p38 mitogen-activated protein kinase and c-Jun-NH2-terminal kinase by BMP-2 and their implication in the stimulation of osteoblastic cell differentiation. J. Bone Miner. Res..

[61] Cai B., Zheng Y., Yan J., Wang J., Liu X., Yin G. (2019). BMP2-mediated PTEN enhancement promotes differentiation of hair follicle stem cells by inducing autophagy. Exp. Cell Res..

[62] Shimizu S., Nakao K., Minato N., Uemoto S. (2015). Innovative Medicine: Basic Research and Development.

[63] Shokouhi B., Coban C., Hasirci V., Aydin E., Dhanasingh A., Shi N., Koyama S., Akira S., Zenke M., Sechi A.S. (2010). The role of multiple toll-like receptor signalling cascades on interactions between biomedical polymers and dendritic cells. Biomaterials.

