# Supplementary material for: Bioactive glass selectively promotes cytotoxicity towards giant cell tumor of bone derived neoplastic stromal cells and induces MAPK signalling dependent autophagy
Source: Bioact Mater. 2022 Feb 28;15:456–68. doi: 10.1016/j.bioactmat.2022.02.021 (PMC8958388; doi:10.1016/j.bioactmat.2022.02.021)
Supplement: Supplemental Table 1 [file mmc1.docx]

| **Supplemental table 1.** Primer used for RT-qPCR analyses | | |
| --- | --- | --- |
| **Gene** | **Forward primer (5´- 3´)** | **Reverse primer (5´- 3´)** |
| ***RPS13*** | GGTTGAAGTTGACATCTGACGA | CTTGTGCAACACCATGTGAAT |
| **RGS2** | caaacagcaagctttcatcaag | cctgaatgcagcaagacca |
| **RSAD2** | atttcccagtggctgaaaac | gtgaccacaggtaatcagatgc |
| **IL6** | gatgagtacaaaagtcctgatcca | ctgcagccactggttctgt |
| **GEM** | AGCACTGGGATTTTCTGGACT | GGGGCTCTTTCTGGACCATC |
| **FOS** | ctaccactcacccgcagact | aggtccgtgcagaagtcct |
| **RGCC** | cagactctaccccagctcttctc | ttctagctcttttgtgtctcctaattt |
| **IFIT2** | agtccagggcttcggataac | tgtgttcacgtaggtcaatgg |
| **OSAL** | GTACCAGCAGAGGGCACG | CCCTGGTCCTCCGGATTTTC |
| **FOSB** | tgtcttcggtggactccttc | gaaggaaccgggcatttc |
| **SPRY2** | GAGTGTTCATCAGCGGGGAA | CACATCTGAACTCCGTGATCG |
| **TMEM158** | cgtaagcccattgagtccac | ccacaccacgatgaccag |
| **BMP2** | cagaccaccggttggaga | ccactcgtttctggtagttcttc |
| **JUN** | ccttctatgacgatgccctc | ccgttgctggactggattat |
| **BPGM** | AGTGCTGTCCTTATGGCAAGA | AGCTTCCTCCATTCCTTCGC |
| **JUND** | CCCCCTTCGGTTCTTTCGAC | AAACAGAAAACCGGGCGAAC |
