# Supplementary figures and images for: Bioactive glass selectively promotes cytotoxicity towards giant cell tumor of bone derived neoplastic stromal cells and induces MAPK signalling dependent autophagy
Source: Bioact Mater. 2022 Feb 28;15:456–68. doi: 10.1016/j.bioactmat.2022.02.021 (PMC8958388; doi:10.1016/j.bioactmat.2022.02.021)

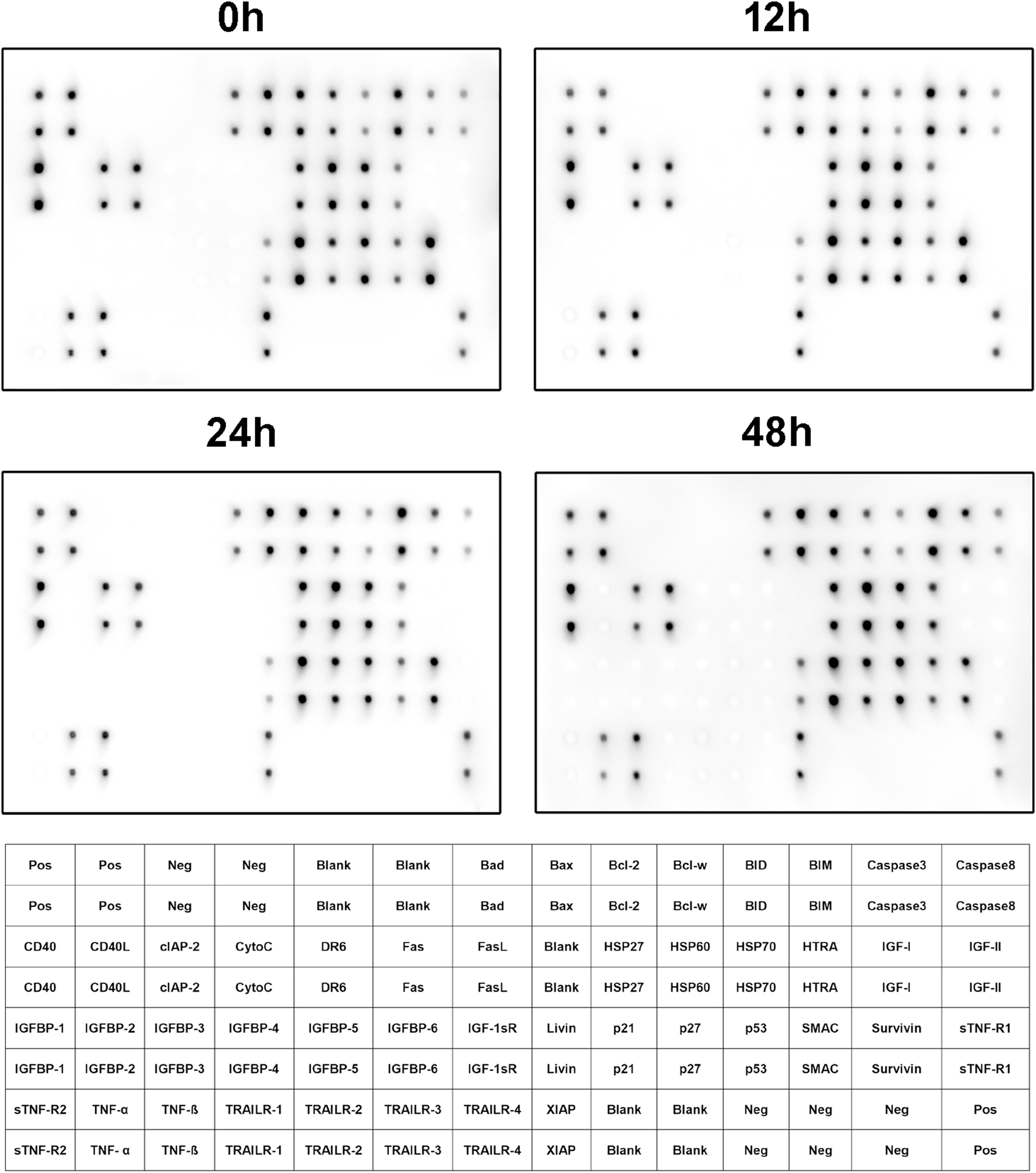

Supplement: Supplemental Fig. 1 [file mmcfigs1.jpg]
